# Risk factors associated with diabetic foot amputation: A retrospective study from a tertiary hospital in Central Malaysia

**DOI:** 10.1371/journal.pone.0335328

**Published:** 2026-06-24

**Authors:** Sanjiv Rampal, Claudia Abreu Lopes, Parichehr Hadi, Vasantha Kumari Neela

**Affiliations:** 1 School of Medicine, International Medical University, Seremban, Malaysia; 2 International Institute for Global Health, United Nations University, Kuala Lumpur, Malaysia; 3 Department of Orthopaedics, Faculty of Medicine and Health Sciences, Universiti Putra Malaysia, Serdang, Malaysia; 4 Department of Medical Microbiology, Faculty of Medicine and Health Sciences, Universiti Putra Malaysia, Serdang, Malaysia; Southern Medical University Nanfang Hospital, CHINA

## Abstract

Diabetes mellitus continues to escalate as a major global health crisis, with diabetic foot infection (DFI) remaining one of the most serious and preventable complications. Despite advances in multidisciplinary care, amputation rates remain high, particularly in low- and middle-income settings. This retrospective study identifies key predictors associated with the level of amputation; major (above the knee) versus minor (at or below the knee) among patients with DFI following surgical decision-making. Electronic medical records of 434 patients admitted with DFI to a tertiary care hospital in Central Malaysia between January 2010 and December 2019 were analysed. Minor amputations accounted for 70.7% of cases, while major amputations comprised the remainder. Most patients (63.8%) presented with advanced disease, with Wagner grade 4 being the most prevalent (40.3%). Binary logistic regression analysis was employed to determine independent predictors of amputation level. Increasing age (OR = 1.06, p = .013) and higher Wagner classification (OR = 15.16, p < .001) emerged as significant independent predictors of major amputation. These results highlight the need for timely intervention and aggressive limb-salvage strategies in high-risk groups.

## Introduction

Diabetic foot infection (DFI) is among the most serious complications of diabetes. Between 15 and 25% of diabetic patients develop a foot ulcer during their lifetime, and up to 70% of such ulcers may become infected, potentially progressing to gangrene and amputation [1]. Minor amputations performed at or below the ankle aim to preserve limb function by removing non-viable tissue early, while major amputations above the ankle are associated with higher mortality and a reduced quality of life [2,3].

The Western Pacific region bears the highest burden of diabetes, accounting for 37% (215 million) of all cases globally [4]. Within this region, Malaysia ranks among the highest-prevalence countries, with 15.6% of adults affected as of 2023, a figure that has risen from 11.2% in 2011 [5]. This upward trend is driven in part by high rates of overweight (32.6%) and obesity (21.8%), and physical inactivity affecting 84% of the adult population [5]. Local studies have begun to characterise the predictors of major amputation: a 10-year retrospective analysis from Hospital Kuala Lumpur identified advanced age (≥65 years) and peripheral arterial disease as key drivers [6], while a study from Pahang implicated treatment non-compliance, low ankle-brachial index, sensory neuropathy, and elevated inflammatory markers including C-reactive protein and creatinine [3]. Despite these contributions, the Malaysian evidence base remains limited.

Several validated wound classification systems have been used to stratify amputation risk in DFI. The Wagner system is of particular relevance: grades 4 and 5, characterized by localized or extensive gangrene secondary to ischemia and infection, carry an increased amputation risk [7]. Delayed presentation of more than 30 days further worsens outcomes, while early surgical debridement has been shown to reduce amputation rates [8]. In LMIC settings including Malaysia, socioeconomic barriers; principally cost of care, transport limitations, and low health literacy contribute to late presentation and a higher likelihood of advanced disease at the time of admission [9,10].

Inflammatory markers are well-established predictors of amputation in DFI. In Malaysia, a retrospective study from Hospital Serdang found that WBC > 12,000/μL and CRP > 80 mg/L were associated with three and 4.3-fold increase in amputation risk, respectively [11]. Peripheral vascular disease (PVD) and neuropathy represent additional critical determinants: a meta-analysis demonstrated that PVD increases amputation risk 4.5-fold and neuropathy 3.2-fold [12]. Multivariate studies have further refined the predictive landscape: among hospitalized DFI patients in the US, PVD, wound necrosis, elevated CRP (>13 mg/L), prior amputation, and inability to tolerate oral intake were identified as independent predictors [13].

Critically, the majority of published studies have focused on predicting whether amputation will occur, rather than what type. This represents a significant evidence gap, particularly in the Asian context where patient demographics, healthcare access, and disease patterns differ from Western cohorts. The present study addresses this gap by identifying predictors of amputation type; major (above the knee) versus minor (at or below the knee) in a cohort of DFI patients who had already undergone amputation at a tertiary care hospital in Central Malaysia. We hypothesize that routinely available variables, including socio-demographic factors, inflammatory markers, and comorbidity profile, can predict amputation type and thus inform perioperative risk stratification and limb-salvage planning.

## Materials and methods

### Study design and procedure

This retrospective cohort study examined electronic medical records (EMR) of patients with DFI who had their feet amputated at a tertiary care hospital in Central Malaysia between January 2010 and December 2019. The primary outcome was the type of amputation: major (above the knee) versus minor (at or below the knee).

Management of diabetic foot infections followed the Malaysian national clinical practice guidelines. Standard care included empirical antibiotic therapy, wound care and dressing, surgical debridement when indicated, and imaging investigations when osteomyelitis was clinically suspected. All patients initially received empirical antibiotic therapy based on the clinical severity of infection. Wound cultures were subsequently obtained, and antimicrobial therapy was adjusted according to microbiological culture and sensitivity results once available. Amputation was performed in cases of non-salvageable infection, as determined by the treating clinical team based on the extent and severity of infection, response to treatment, and microbiological findings.

Peripheral vascular disease and peripheral neuropathy were diagnosed clinically based on standard assessments documented in medical records. As part of the routine evaluation of the diabetic foot, peripheral neuropathy was assessed using the Semmes-Weinstein Monofilament Test to identify patients with a loss of protective sensation (LOPS). The test consists of using a 10g monofilament to apply pressure to specific sites on the foot. The filament is pressed against the skin until it bows; a lack of sensation at any site indicates a high-risk foot. A diagnosis was established based on clinical findings indicative of reduced sensation, which, combined with vascular status, categorized the patient as having a “foot at risk” for ulceration or further complications. This designation follows the IWGDF (International Working Group on the Diabetic Foot) risk stratification, where patients with loss of sensation, peripheral artery disease, or previous foot deformities are categorized by their likelihood of future amputation.

Peripheral vascular disease (PVD) was assessed as part of routine clinical evaluation, particularly in patients presenting with diabetic foot infections. This included a comprehensive examination of arterial supply and peripheral perfusion in the lower limbs. A diagnosis of PVD was established based on clinical evidence of impaired blood flow and a documented Ankle-Brachial Index (ABI) of less than 0.5, indicating significant arterial insufficiency.

The severity of diabetic foot lesions was classified using the Wagner classification system. Wagner grading was assigned by the attending specialist as part of a multidisciplinary orthopedic diabetic foot team comprising approximately 15 clinicians with varying levels of clinical experience.

### Inclusion and exclusion criteria

The study included adult patients (≥18 years) with diabetes mellitus who were hospitalized for lower limb amputation due to DFI at a hospital in Kuala Lumpur between January 2010 and December 2019. Eligible patients had DFI documented as the primary diagnosis in the electronic medical record. Both insulin-treated and non–insulin-treated patients were included in the study population. Patients were excluded if they were pregnant, younger than 18 years of age. Patients admitted for other causes of foot infection not related to DFI such as dermatitis or dry gangrene were also excluded. After applying the inclusion and exclusion criteria, a total of 434 patients were included in the final dataset. Among these patients, 34.6% were receiving insulin therapy at the time of admission.

### Data handling and statistical analysis

The dataset was extracted from the electronic medical record (EMR) system and included routinely collected clinical information (medical history, medications, laboratory results, and clinician notes) relevant to the study objectives. Only variables pertinent to the analysis were included in the extracted dataset. Prior to transferring to the Principal Investigator, the dataset was fully de-identified, with all personal identifiers removed and replaced by unique random study identifiers.

Data cleaning was performed prior to analysis and included removal of duplicate records, harmonization of variable coding, and validation of value ranges. The extent of missing data was assessed across variables; analyses were conducted using a complete case approach for each model.

The dataset included demographic and clinical variables such as age, sex, ethnicity, smoking status, Wagner grade, inflammatory markers (erythrocyte sedimentation rate [ESR], white blood cell count [WBC], C-reactive protein [CRP]), peripheral neuropathy, peripheral vascular disease (PVD), osteomyelitis, gangrene, and amputation history and type. Amputations were classified as major (above the knee) or minor (at or below the knee). Variables not routinely captured in the EMR, including ulcer duration prior to admission and imaging findings (X-ray or magnetic resonance imaging), were not available for analysis.

Descriptive statistics were used to summarise the data. Continuous variables were reported as mean and standard deviation (SD) or median and interquartile range (IQR), depending on distribution, while categorical variables were presented as frequencies and percentages.

Bivariate analyses were conducted to assess associations between risk factors and amputation type. The Mann–Whitney U test was used for continuous variables, and the chi-square test (or Fisher’s exact test where appropriate) for categorical variables. Comparisons of proportions between groups were further explored using z-tests where relevant.

Multivariable analysis was performed using binary logistic regression to identify factors independently associated with amputation type. Variables with a statistically significant association in bivariate analysis (p < 0.05) were included in the model. Results were reported as odds ratios (OR) with 95% confidence intervals (CI). Statistical significance was defined as a two-sided p-value < 0.05. All data preparation, analysis, and visualisation were conducted using R (RStudio). The dataset is available at OpenICPSR repository [14], and the analysis code is accessible via RPubs [15].

## Results

### Sociodemographic characteristics

**Fig 1:**
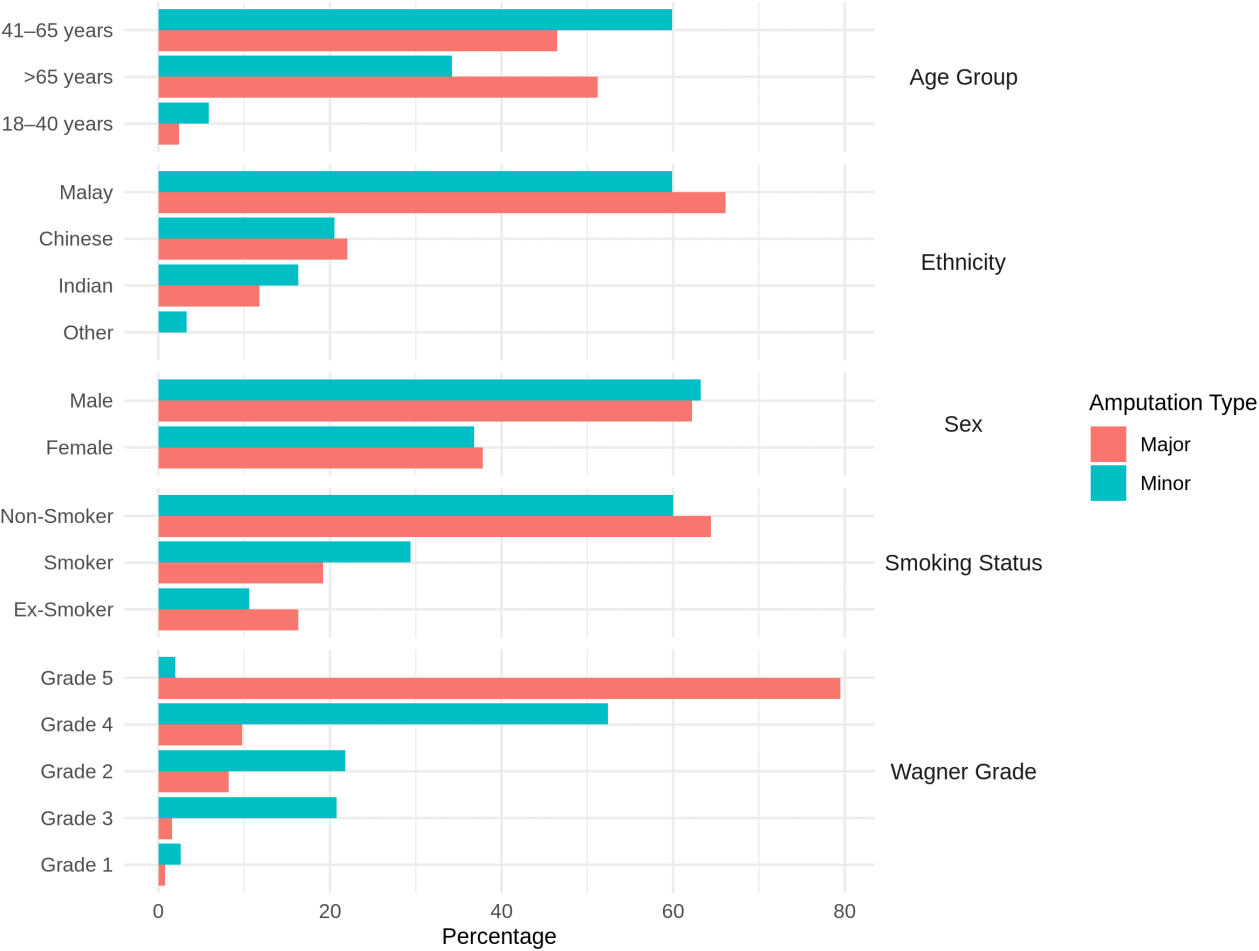
Percentage distribution of individuals across demographic groups, stratified by amputation type.

The socio-demographic characteristics of DFI patients at a hospital in Kuala Lumpur are shown in Fig 1. More than half of the patients (56%) are aged between 41 and 65 years, followed by those over 65 (39.2%), with 4.8% being between 18 and 40. Among the patients, 62.9% were male and 37.1% were female. Across all major ethnic groups in Malaysia (Malay, Indian, and Chinese), the Indian patients had the lowest representation at 15%, while Malay patients were the most represented at 61.8%. Additionally, the majority (61.2%) of the patients were found to be non-smokers. All demographic information was complete except for smoking status that contained 15% of missing values.

The breakdown of socio-demographic characteristics by type of amputation is also presented in Fig 1. For 59.4% of patients, this was their first amputation while 40.6% of patients had the history of amputation. Most patients had minor amputations (n = 307; 70.7%), while 29.3% (n:127) had major amputations. According to the chi-square test of independence, age group was found to be significantly associated with the type of amputation (*p* = .003). These findings indicate that major amputations above the knee were more common among elderly patients aged ≥65 (51.2%). In contrast, minor amputations below or at the level of the knee were more common among patients aged 41–65 (59.9%).

### Indicators of inflammation

Elevated levels of markers measuring the inflammatory response in diabetic foot infection patients such as C-reactive protein (CRP), erythrocyte sedimentation rate (ESR), procalcitonin (PCT), and white blood cell count (WBC) indicate the presence and severity of infection.

Since the inflammation markers present a skewed distribution, the Mann-Whitney test was used to compare the mean difference between the two types of amputation (Table 1). The results showed that WBC, CRP, and ESR median levels differ significantly by type of amputation. Across all markers, patients with major amputations showed higher levels of these inflammation markers.

**Table 1:** Association between inflammation markers and amputation (Mann-Whitney U test).

Markers	Missing	Minor, median (IQR)	Major, median (IQR)	*p*-value
WBC	<1%	15.5 (9.22) x 10³/μL	18 (9.15) x 10³/μL	<.001***
CRP	47.5%	139 (172) mg/L	191 (123) mg/L	.002**
ESR	36.9%	101 (28) mm/hr	108 (29) mm/hr	.021*

*Note.* IQR = Interquartile range. Significance: *p <.05, **p <.01, ***p <.001.

### Complications from diabetes

The duration of diabetes varies between 1 and 37 years with mean ± SD of 10.95 ± 8.1 years (the percentage of missing values is 37.1% of patients). The most frequent diabetic complications were gangrene (66.4%), osteomyelitis (64.2%), and peripheral neuropathy (25.3%) followed by peripheral vascular disease (13.8%). The test of association between amputation and complications from diabetes revealed that all complications were significantly associated with the type of amputation (Table 2).

**Table 2:** Association between complications from diabetes and amputation categories (Two proportion z-test, row percentages).

Complications	Frequency (%)	Minor, n (%)	Major, n (%)	*p*-value
Gangrene	188 (66.4%)	133 (70.7%)	55 (29.3%)	<.001***
Peripheral Neuropathy	110 (25.3%)	87 (79.1%)	23 (20.9%)	<.001***
Osteomyelitis	185 (64.2%)	139 (75.1%)	46 (24.9%)	<.001***
PVD	60 (13.8%)	36 (60%)	24 (40%)	<.05*

*Note.* n = sample size. Significance: *p <.05, **p <.01, ***p <.001.

Wagner grades 4 and 5 are the most common (40.3% and 23.5%, respectively). The results in Table 3 indicate a significant association between Wagner classification and amputation [X2(4) =288.90, p < .001]. The two-proportion z-test is used here to compare the proportion of major versus minor amputations within each Wagner grade. Grades 1–4 often resulted in minor amputations, while major amputations were mainly performed in grade 5 (79.5% of major amputations).

**Table 3:** Association between Wagner grade and type of amputation (Two-proportion z-test, column percentages, missing = 0%).

Level	Frequency (%)	Minor, n (%)	Major, n (%)	z-statistic	*p*-value
Grade 1	9 (2.1%)	8 (2.6%)	1 (0.8%)	3.30	<.001***
Grade 2	77 (18%)	67 (21.8%)	10 (8.2%)	9.19	<.001***
Grade 3	66 (15.4%)	64 (20.8%)	2 (1.6%)	10.79	<.001***
Grade 4	173 (40.3%)	161 (52.4%)	12 (9.8%)	16.02	<.001***
Grade 5	105 (24.0%)	6 (2.0%)	97 (79.5%)	–12.68	<.001***

*Note.* n = sample size. Significance: *p <.05, **p <.01, ***p <.001.

### Binary logistic model

The multivariable binary logistic regression analysis to predict minor vs major amputation including age, WBC abnormalities, Wagner grade, osteomyelitis, peripheral neuropathy, PVD, and duration of diabetes as predictors (Table 4). The overall model fit was assessed using the Akaike Information Criterion (AIC) indicating the relative goodness of fit of the model (AIC = 206.87).

**Table 4:** Binary logistic regression predicting odds of major (vs minor) amputation (AIC = 206.87).

Predictor	Estimate	SE	OR	95% CI (Lower)	95% CI (Upper)	z	*p*-value
(Intercept)	−15.53	2.68	<.01	<.01	<.01	−5.81	<.001 ***
Age (years)	0.06	0.023	1.06	1.01	1.11	2.49	.013 *
WBC < 4 (abnormal)	−13.20	1455.40	<.01	–	<.01	−0.01	.993
WBC > 11 (abnormal)	0.55	0.73	1.81	0.46	8.71	0.81	.418
Wagner grade (1–5)	2.72	0.47	15.16	6.69	41.68	5.85	<.001 ***
Osteomyelitis (yes)	−0.13	0.51	0.88	0.32	2.43	−0.251	.802
Peripheral neuropathy (yes)	−1.00	0.60	0.37	0.11	1.15	−1.655	.098
Peripheral vascular disease (yes)	−0.35	0.73	0.71	0.16	2.81	−0.481	.631
Duration of diabetes (years)	−0.03	0.04	0.97	0.91	1.04	−0.750	.453

*Note.* B = unstandardised coefficient, SE = standard error, OR = odds ratio, CI = confidence interval, Significance: ***p < .001.

Age and Wagner grade emerged as significant predictors of major amputation. Increasing age was associated with higher odds of major amputation (OR = 1.06, 95% CI: 1.01–1.11, p = .013), indicating that each additional year of age increased the likelihood of major amputation by approximately 6%. Wagner grade showed an association with amputation severity (OR = 15.16, 95% CI: 6.69–41.68, p < .001), suggesting that patients presenting with more advanced ulcer grades were more likely to undergo major amputation. Peripheral neuropathy showed a trend toward reduced odds of major amputation, although this association did not reach statistical significance (p = .098).

The model’s explanatory power was evaluated using pseudo-R² statistics, indicating the proportion of variance in amputation severity explained by the predictors. The McFadden’s Pseudo R² = 0.636 and the Cragg-Uhler R² = 0.893, denoted that the model has a strong explanatory power.

## Discussion

This study examined clinical and demographic characteristics associated with the severity of amputation among patients hospitalized with DFI in a tertiary hospital in Malaysia. Although the findings derive from a single-centre dataset, they are consistent with results reported in other Malaysian hospital-based studies [16–20], which strengthens the plausibility of the observed patterns. Nevertheless, confirmation through larger multi-centre studies would further validate these findings.

Older age emerged as a significant predictor of major amputation. Age may influence amputation risk through several mechanisms, including longer duration of diabetes, higher prevalence of vascular complications, and delayed wound healing. In this study, 51.2% of major amputations occurred among individuals aged ≥65 years, while the most common age group among hospitalized DFI patients overall was 41–65 years. These findings align with previous studies reporting that DFI and amputation risk increase with age. For example, a study from Ethiopia found that most DFI patients were aged 58–67 years [21], while other research conducted in Malaysia reported a mean age of 58.6 years among DFI patients [16]. However, not all studies report a similar association; for instance, research from India found no statistically significant relationship between age and the type of amputation [22], suggesting that contextual or healthcare system factors may also influence outcomes.

In terms of gender, most patients in this study were male (62.9%), consistent with previous Malaysian studies reporting similar gender distributions among DFI patients [18,19]. Although male patients were more frequently affected by DFI, gender was not significantly associated with the likelihood of undergoing major versus minor amputation. This finding is consistent with other studies that did not identify gender as a significant predictor of amputation severity [9,23]. However, the literature remains mixed, with some studies identifying male gender as a risk factor for amputation [9,24,25]. Differences in occupational exposure, health-seeking behaviour, and access to routine diabetic care have been proposed as potential explanations for these variations across settings.

The ethnic distribution of patients in this study reflected the broader population structure of Malaysia, with Malays accounting for the largest proportion of cases, followed by Chinese, and Indians. Similar patterns have been reported in other Malaysian hospital-based studies, including research conducted at Hospital Segamat in Johor and in the state of Pahang, where Malays constituted the majority of DFI patients [20,26]. However, while Malays represent the largest proportion of cases due to population distribution, previous research has suggested that Indians may have a higher risk of diabetes-related complications, including DFI-related amputations [17]. In the present study, ethnicity was not significantly associated with the risk of major versus minor amputation.

Smoking, although not significantly different between the major and minor amputation groups in this study, remains an established risk factor for diabetic foot complications and lower-limb amputation due to its association with impaired vascular function and delayed wound healing [27].

In the binary logistic regression analysis, only Wagner grade and age remained significant independent predictors of major versus minor amputation (p < 0.0001). Elevated WBC levels and diabetes-related complications were not independently associated with amputation severity after adjustment. This attenuation likely reflects the clustering of advanced ulcer grades, inflammatory markers, and diabetes-related complications among older patients and those with longer disease duration.

The significant association between higher Wagner grades and major amputation observed in this study is consistent with previous research demonstrating that advanced ulcer severity is a major determinant of amputation risk [28–30]. Wagner grade 4 and 5 ulcers represent advanced stages of tissue damage, frequently involving gangrene and deep infection, which increases the likelihood of extensive surgical intervention.

These results have direct clinical implications. Routine Wagner grading at admission, combined with age-based risk stratification, can help clinicians identify patients at highest risk of major amputation early in the management pathway enabling timely escalation to specialist limb-salvage services, targeted vascular assessment, and informed perioperative planning. Given Malaysia’s lack of a standardised national diabetic foot screening programme, embedding such stratification into routine tertiary care protocols could meaningfully reduce the burden of major amputation.

This study has several limitations. First, it was conducted at a single tertiary hospital in Central Malaysia, limiting generalisability. Malaysia’s dual public-private healthcare system, with surgical theatre access concentrated in state capital hospitals and variable specialist referral pathways between rural and urban areas, likely contributed to increased morbidity in this cohort. Second, only DFI patients who underwent amputation were included, so results apply specifically to those requiring surgical intervention rather than all DFI patients at risk of amputation.

Third, inflammatory markers were limited to WBC, CRP, and ESR; other biomarkers such as cytokines were unavailable. Finally, potentially important confounders including HbA1c levels (investigated in <25% of patients), socioeconomic status, and presentation delay could not be comprehensively analysed due to incomplete data. This reflected poor standardization of protocols across referring facilities, interpersonal differences in care delivery, and challenges accessing income data due to Personal Data Protection Act requirements. These factors should be considered when interpreting the findings. Future prospective and multi-centre studies incorporating HbA1c, vascular imaging, and socioeconomic data are warranted to validate and extend these findings across the broader Malaysian healthcare system.

## Conclusion

In conclusion, this retrospective study of 434 patients with DFI revealed that advanced Wagner grade (grades 4–5) and older age (≥65 years) emerged as the two independent predictors of major versus minor amputation in a Malaysian tertiary hospital. A key strength of this study is that it focused exclusively on patients who have already undergone amputation, thereby isolating the factors that distinguish amputation level rather than the decision to amputate. The findings from this Central Malaysian cohort are particularly valuable given that the Western Pacific context characterized by a high diabetes burden, late disease presentation, and systemic healthcare access constraints differs from the Western settings that dominate the existing evidence base.
