# Supplementary material for: Risk factors associated with diabetic foot amputation: A retrospective study from a tertiary hospital in Central Malaysia
Source: PLoS One. 2026 Jun 24;21(6):e0335328. doi: 10.1371/journal.pone.0335328 (PMC13293416; doi:10.1371/journal.pone.0335328)
Supplement: Attachment — Submitted filename: Response_Reviewer3.docx [file pone.0335328.s002.docx]

**Response to Reviewer 3**

We sincerely thank Reviewer 3 for the thorough and constructive feedback. The comments have helped us substantially improve the clarity, structure, and scientific rigor of the manuscript. We address each point below.

| # | Comment | Response |
| --- | --- | --- |
| 1 | Nonacademic words (silent epidemic, insight), lack of study design, clarify that the study from single center | We thank the reviewer for this important observation. Nonacademic terms such as “silent epidemic” and “insight” have been removed and replaced with more appropriate scientific language (e.g., “rising burden”). We have also explicitly stated the study design in the Methods section. Furthermore, we clarified that this is a single-center study, specifying that the population comprises patients hospitalized for diabetic foot infection (DFI)-related amputation in a single hospital over a 10-year period. |
|  | Abstract |  |
| 2 | Correct the format. | The Abstract has been reformatted in accordance with journal guidelines. |
| 3 | Add the definition of major and minor amputation | We have added clear definitions in the Methods section. Major amputation is defined as amputation above the knee, while minor amputation is defined as amputation at or below the level of the knee. |
| 4 | Add more details abouts statistical results such as p value | We have added p-values to all relevant tables and summarized key statistical findings in the Abstract. |
| 5 | Avoid general terms and nonacademic terms | The manuscript has been carefully revised to remove nonacademic and overly general language throughout. |
| 6 | Conclusion should be more descriptive than interpretative, and should be more concise and precise | The Abstract conclusion has been rewritten to be more concise, descriptive, and aligned with the study findings. |
|  | Introduction |  |
| 7 | Overly long, and a lot of repetition | The Introduction has been substantially shortened and redundant content removed. |
| 8 | Have a lot of information about DM, not necessary to the scope of paper, focus on DFI | We have removed excessive background on diabetes mellitus and refocused the Introduction specifically on diabetic foot infection (DFI) and its clinical relevance. |
| 9 | Lacks of study flow toward aim | The Introduction has been reorganized to improve logical flow, culminating clearly in the study objective. |
| 10 | Clear the knowledge gap and objective | We have refined the study objective and explicitly stated the knowledge gap. Specifically, this study uniquely distinguishes predictors of major versus minor amputation after the clinical decision to amputate has been made, whereas prior studies primarily focus on risk factors for amputation occurrence. |
|  | Method |  |
| 11 | No need to mention detailed local administration rules | We acknowledge this point. However, this information was included to comply with journal requirements. To improve readability, it has been relocated to a dedicated subsection (Ethics and Data Governance). |
| 12 | Lacks of inclusion and exclusion criteria | Detailed inclusion and exclusion criteria have now been added to the Methods section. |
| 13 | Lacks of detailed data handling (incomplete data, deaths, conversion from minor to major amputation, recurrent cases, bilateral) | We appreciate this comment. Due to dataset limitations, detailed information was not available for several of these variables. However, we have included available information on recurrent cases, indicating that 59.4% were first amputations and 40.6% had a prior history of amputation. This limitation has been acknowledged in the manuscript. |
| 14 | Inconsistent definitions (major and minor amputation according to literature | We have standardized definitions in accordance with the literature: major amputation (above the knee) and minor amputation (at or below the knee level). |
| 15 | Peripheral neuropathy and peripheral vascular disease, way of diagnosis?? | We have clarified that these conditions were clinically diagnosed based on documentation in the patients’ medical records. |
| 16 | Ethical approval should be more concise | To address this, we have streamlined the presentation and moved detailed information into a dedicated subsection (Ethics and Data Governance) in accordance with journal requirements. |
|  | Results |  |
| 17 | Results should only report findings, without explanation and interpretation | The Results section has been revised to present findings only, without interpretation. |
| 18 | Make more concise without addition of literature and background information | The Results section has been streamlined, and background or literature references have been removed. |
|  | Discussion: |  |
| 19 | Too long, a lot of repetition, need to be concise | The Discussion has been shortened and redundant content removed. |
| 20 | Studies put back to back, need to incorporate its implication to study results | We have revised the Discussion to better integrate existing literature and explicitly relate prior findings to our results. |
| 21 | Make it more clear | The Discussion has been restructured to improve clarity and readability. |
| 22 | Results should be interpreted and supported with literature | We have strengthened the Discussion by linking our findings to relevant literature and providing clearer interpretation. |
|  | Limitation |  |
| 23 | Should be addressed, and mentioned after discussion | The Limitations section has been rewritten and positioned immediately after the Discussion, in line with standard structure. |
|  | Conclusion |  |
| 24 | Should be more concise and highlight the contribution of the study | The Conclusion has been revised to be more concise and to clearly emphasize the study’s key contribution, namely, identifying predictors of amputation level (major vs. minor) among patients already selected for amputation. |
