# Supplementary material for: Risk factors associated with diabetic foot amputation: A retrospective study from a tertiary hospital in Central Malaysia
Source: PLoS One. 2026 Jun 24;21(6):e0335328. doi: 10.1371/journal.pone.0335328 (PMC13293416; doi:10.1371/journal.pone.0335328)
Supplement: Attachment — Submitted filename: Reviewer3.odt [file pone.0335328.s003.odt]

Reviewer #3:
Comments
Response
Title: Use neutral language
Changed to: Risk Factors for Diabetic Foot Amputation: Evidence from Central Malaysia
Abstract: Use neutral language, sharpen the study gap, add p value
We improved the neutrality of the language, added a study gap and added p values to the abstract. ‘This retrospective study aimed to identify key predictors associated with the level of amputation—major (above the knee) versus minor (at or below the knee)—among patients with DFI following surgical decision-making. In doing so, it addresses a gap in the literature, which has largely focused on predicting the occurrence of amputation rather than the level at which it is performed’
Introduction: Still too long, and a lot of repetition, Research gap not clearly identified, Flow must be improved
The introduction was re-written to be sharper, shorter and to improve the overall flow. The research gap was identified as ‘The present study addresses this gap by identifying predictors of amputation type—major (above the knee) versus minor (at or below the knee)—in a cohort of DFI patients who had already undergone amputation at a tertiary centre in Central Malaysia. Unlike most prior studies, our analysis is restricted to amputated patients, thereby isolating the clinical and demographic variables that distinguish amputation level rather than the decision to amputate.’
Method: Clearly define the primary outcome, Lacks of detailed data handling, Diagnosis of Peripheral neuropathy and peripheral vascular disease (PVD) not objective
The primary outcome was added to the study design: The primary outcome was the type of amputation: major (above the knee) versus minor (at or below the knee).
Data handling is described in the Dataset section (more detail added). This paragraph was added to elucidate the diagnosis of PN and PVD ‘Peripheral neuropathy and peripheral vascular disease were diagnosed clinically based on standard assessments documented in the medical records. As part of routine evaluation in patients with diabetic foot, peripheral neuropathy was assessed through examination of lower limb sensory and neurological function. A diagnosis was made based on clinical findings indicative of reduced sensation.
Peripheral vascular disease was similarly assessed as part of routine clinical evaluation, particularly in patients presenting with diabetic foot infections. This included assessment of arterial supply and peripheral perfusion in the lower limbs. A diagnosis was established based on clinical evidence of impaired blood flow.’
Results: Mixing results with interpretation
The results were reviewed to include only results
Limitation: Overly long
The limitations section was re-written to make it more concise
Conclusion: should be more concise and highlight the contribution of the study
The conclusion was re-written as the conclusion of the study stated as: ‘A key strength of this study is its focus exclusively on patients who have already undergone amputation, thereby isolating the factors that distinguish amputation level rather than the decision to amputate. This approach addresses a specific and underexplored evidence gap in the DFI literature, where most published studies conflate the prediction of amputation occurrence with the prediction of amputation severity. The findings from this Central Malaysian cohort are particularly valuable given that the Southeast Asian context—characterised by a high diabetes burden, late disease presentation, and systemic healthcare access constraints—differs substantially from the Western settings that dominate the existing evidence base.’
